# Aqueous extract of bay leaf (*Laurus nobilis*) ameliorates testicular toxicity induced by aluminum chloride in rats

**DOI:** 10.14202/vetworld.2022.2525-2534

**Published:** 2022-11-09

**Authors:** Ayodeji O. Falade, Kayode E. Adewole, Abdul-Rahman O. Adekola, Hilary A. Ikokoh, Kunle Okaiyeto, Oluwafemi O. Oguntibeju

**Affiliations:** 1 Department of Biochemistry, Faculty of Basic Medical Sciences, University of Medical Sciences, Ondo 351101, Ondo State, Nigeria; 2 Phytomedicine and Phytochemistry Group, Department of Biomedical Sciences, Faculty of Health and Wellness Sciences, Cape Peninsula University of Technology, Bellville 7535, South Africa

**Keywords:** anti-inflammatory property, reproductive toxicology, sperm parameters, spices, testicular enzymes

## Abstract

**Background and Aim:**

Human exposure to aluminum is inevitable, and one of the most adverse health effects of aluminum is a decrease in male fertility rates. Therefore, this study investigated the ameliorative effects of an aqueous extract from *Laurus nobilis-*bay leaf (BL) on aluminum chloride (AlCl_3_)-induced testicular toxicity in rats.

**Materials and Methods:**

Twenty-four Wistar rats were divided into four groups (n = 6, each group): The control (group 1) received normal saline; Group 2 animals were intraperitoneally administered with 30 mg/kg body weight (BW) AlCl_3_; and Groups 3 and 4 were co-administered AlCl_3_ with 125 or 250 mg/kg BW of BL extract, respectively, for 21 days. Testes, epididymis, and blood samples were collected. Testicular plasma enzyme activity was measured using a spectrophotometric assay, while concentrations of inflammatory biomarkers were determined using enzyme-linked immunosorbent assay kits.

**Results:**

There was a significant increase (p < 0.05) in testicular enzyme activity in the group treated with AlCl_3_. However, there was no significant (p > 0.05) difference in testicular enzyme activity in groups co-administered AlCl_3_ and BL extract as compared with that in control. There was a significant (p *<* 0.05) increase in testicular nitrite concentration in the AlCl_3_-treated group, whereas the administration of BL extract significantly (p < 0.05) decreased nitrite concentration in Groups 3 and 4. Furthermore, the administration of BL extracts increased sperm count and improved the morphology of the testes in AlCl_3_-treated rats. Flavonoids, phenolic compounds, alkaloids, tannin, glycosides, saponin, anthraquinones, and steroids were identified in BL extract, with alkaloids and glycosides being the most abundant.

**Conclusion:**

Aqueous extract from BL ameliorated the toxic effect of AlCl_3_ and exhibited anti-inflammatory properties by inhibiting nitrite production while improving sperm count and morphology in AlCl_3_-treated rats. The bioactivity of the extract may be attributed to the presence of a wide range of phytochemicals. Therefore, BL aqueous extract could be a promising source of novel compounds with male fertility-promoting and anti-inflammatory properties.

## Introduction

Metals are ubiquitous and have multiple industrial uses [1]. They persist in nature for long, making it difficult for humans to avoid metal exposure [2]. Aluminum is found in airborne dust particles, food additives, phosphate binders, antacids, buffered analgesics, antiperspirants, cooking vessels, tinned boxes, infant formulas, cosmetics, mining, drinking water, and several herbal preparations [1, 3]. For example, aluminum chloride (AlCl_3_) is present in many manufactured consumables and is considered a toxic element [4]. Heavy metals are regarded as harmful compounds that pose health risks to humans and animals, as frequent ingestion of small amounts of these metals causes their progressive and irreversible accumulation [5]. Recently, the detrimental health impacts of aluminum found in drinking water have been identified as a possible concern to public health [6, 7]. Continuous exposure to aluminum from various sources may cause accumulation in several tissues [8], inducing oxidative stress and lipid peroxidation, which triggers several biochemical and physiological dysfunctions [9, 10], such as neurotoxicity, hepatotoxicity, nephrotoxicity, cardiotoxicity, and reproductive toxicity [11–13],

One of the most conspicuous health effects of aluminum is a decrease in male fertility rates [1]. For example, Berihu [14] and Pandey and Jain [15] reported that aluminum accumulation causes male reproductive toxicity through various processes, including inducing oxidative stress, interfering with spermatogenesis and steroidogenesis, altering cell communication, disrupting the blood-testis barrier, and influencing the endocrine system. Consumption of aluminum-containing items raises the concentration of this metallic element in consumers’ organs, resulting in testicular tissue injuries. Aluminum chloride has been shown to cause testicular toxicity in adult male Wistar rats [4] and cause male reproductive impairment in rats [16]_._ Aluminum has also been linked to testicular damage caused by increased nitric oxide (NO) levels [17]. Several studies have found that aluminum depletes the antioxidant capacity of cells while increasing lipid peroxidation [5, 18–20]. Furthermore, high levels of aluminum in human spermatozoa and seminal plasma reduce sperm viability and motility [21]. Both male rats and mice with testicular aluminum buildup experienced spermatocyte/spermatid necrosis and a significant decline in fertility [17]. One study has revealed that exposure to aluminum at 100 mg Al^3+^/kg body weight (BW)/day induces inflammation, free radical production, and lipid peroxidation [22].

Traditional medicine is a promising medical alternative that uses plant extracts containing biologically active compounds to treat various chronic infections and diseases [23]. Herbal supplements have been the focus of considerable research because of their potential effectiveness, low cost, and minimal toxicity [24]. The laurel tree is an economically important medicinal plant that contains several phytochemicals with remarkable therapeutic benefits to humans [25]. The dried leaves of *Laurus nobilis* are used in the culinary and food industries as a spice or flavoring ingredient [26]. *L. nobilis*, generally known as bay, is a native Mediterranean evergreen shrub that is widely grown in West Asia, South Europe, North Africa, and America [27]. The therapeutic potential of *L. nobilis* has been widely reported [28–33]. However, despite numerous studies on *L. nobilis*, there is a paucity of information regarding its protective role in male infertility induced by aluminum toxicity.

Therefore, this study sought to explore the ameliorative effect of an aqueous extract of *L. nobilis* against aluminum chloride-induced testicular toxicity in male Wistar rats.

## Materials and Methods

### Ethical approval

The animals used for this study were given humane care as approved by the Ethical Approval Committee of the University of Medical Sciences Ondo, Ondo State, Nigeria (EAC14/2021).

### Study period and location

The study was conducted from April 2021 to March 2022 at the Department of Biochemistry, Faculty of Basic Medical Sciences, University of Medical Sciences Ondo, Ondo State, Nigeria.

### Chemicals

Aluminum chloride was obtained from Guangdong Scientific Technical Company Ltd. (Guangdong, China); “acid phosphatase (ACP), lactate dehydrogenase (LDH), alkaline phosphatase (ALP), and glucose-6-phosphate dehydrogenase (G-6-PDH) assay kits were obtained from Fortress Diagnostics Limited, Antrim, United Kingdom, while enzyme-linked immunosorbent assay (ELISA) kits were sourced from BioLegend Inc., San Diego, USA. All other chemicals used were of analytical grade and water was glass distilled.”

### Sample collection and preparation

Bay leaf (BL) samples were obtained at Shoprite Mall, Akure, Ondo State, Nigeria. The samples were subsequently validated at the Centre for Research and Development, Federal University of Technology, Akure, and deposited at the University herbarium with voucher number 0299. The samples were then milled into a fine powder using an electric blender, and 500 g of the milled sample was dispensed in 2 L of distilled water (250 g/mL), vigorously shaken, and left standing for 24 h [34]. Subsequently, the mixture was filtered using a vacuum pump (WenlingTingwei Vacuum Equipment Co. Ltd., Zhejiang, China) and concentrated using a TT-420 thermostatic water bath (Techmel Instruments, USA) at 60°C for 5 days. The concentrate obtained (aqueous extract) was stored in ice-cold conditions in a freezer (−4°C).

### Qualitative phytochemical analysis

The aqueous extract of BL was screened for the presence of different phytochemicals, including saponins [35], tannins, terpenoids [35], phenolic compounds [36], flavonoids [37], glycosides [35], steroids [35], anthraquinones, and alkaloids [38]. To test for saponins, 5 mL of distilled water was added to 1 mL of the working extract and mixed vigorously; consistent frothing confirms the presence of saponins [35]. To test for tannin, 1.5 g of ferric chloride was dissolved in 10 mL of distilled water, and then, 1 mL of extract, in a beaker, was placed in a water bath for 5 min at 100°C after which two drops of ferric chloride solution were added. The formation of a blue-black color confirms the presence of tannins [35]. To test for phenolic compounds, 0.5 g of ferric chloride was added to 10 mL of distilled water, after which three drops of 0.5 g of ferric chloride solution were added to the 1 mL of the aqueous extract; a greenish-black color confirms the presence of phenolic compounds [36]. To test for flavonoids, 1.0 mL of 10% w/v NaOH was added to 3.0 mL of the aqueous extract; a yellow color confirms the presence of flavonoids [37]. To test for glycosides (Kiliani’s test), 1 mL of aqueous extract was added to 2 mL of glacial acetic acid, after which one drop of 1.5 g of ferric chloride dissolved in 10 mL was added. Concentrated H_2_SO_4_ (1 mL) was then added, and the formation of brown color at the interface confirmed the presence of glycosides [35]. To test for steroids, 1 mL of the working extract was mixed with 2 mL of acetic acid, and 2 mL of concentrated H_2_SO_4_ was added. A color change from violet to blue confirms the presence of steroids [35]. To test for alkaloids, 0.1 g of picric acid was added to 10 mL of distilled water to form a solution known as Hager’s reagent. An aqueous extract (1 mL) was added to the Hager’s reagent (2 mL) and mixed thoroughly. A yellow precipitate confirms the presence of alkaloids [38]. To test for anthraquinones, 5 mL of the aqueous extract was mixed with 10 mL of benzene and the mixture was filtered; 5 mL of 10% ammonia was added to the resulting filtrate and the mixture was shaken. The appearance of pink color confirms the presence of anthraquinones [38].

### Animal grouping and treatment

Twenty-four male Wistar rats (130–190 g) were sourced from the Department of Biochemistry, Federal University of Technology, Akure, Ondo State, and acclimatized for 4 weeks at the animal house of the University of Medical Sciences, Ondo City, Ondo State, Nigeria. During acclimatization, the animals were fed a commercial diet and water *ad libitum* before the commencement of the experiment. Aluminum chloride was dissolved in normal saline and administered intraperitoneally (i.p.) at 30 mg/kg BW [39], while BL extract was dissolved in distilled water and administered orally at 125 and 250 mg/kg BW [40]. The rats were randomly divided into four treatment groups of six rats as follows:

- Group 1 (Control): Rats administered normal saline orally
- Group 2 (AlCl_3_): Rats administered AlCl_3_ (30 mg/kg BW, i.p.)
- Group 3 (AlCl_3_ + 125mg/kg BL): Rats co-administered AlCl_3_ (30 mg/kg BW, i.p.) and BL aqueous extract (125 mg/kg BW, orally)
- Group 4 (AlCl_3_ + 250 mg/kg BL): Rats co-administered AlCl_3_ (30 mg/kg BW, i.p.) and BL aqueous extract (250 mg/kg BW, orally).

The treatment lasted 21 days, after which the animals were euthanized through cervical dislocation under anesthesia, and testes, epididymis, and blood samples were collected for biochemical analyses. The rat testes were weighed and homogenized in ice-cold phosphate buffer (0.1 M, pH 7.4) using a DEL-1328 electric homogenizer; thereafter, the homogenized samples were centrifuged at 10,000× *g* for 15 min using an MV 16RF cold centrifuge (Inspiration Marvotech); the recovered supernatant from testis homogenate was collected and used to estimate the level of inflammatory biomarkers: Nitrite, tumor necrosis factor-alpha (TNF-α), and interleukin-6 (IL-6).

### Testicular enzyme assays

#### Determination of ALP activity

Alkaline phosphatase activity was determined according to the manufacturer’s manual. Briefly, 0.05 mL of sample was added to 0.5mL of the reagent (sodium thymolphthalein monophosphate + 2-amino-2-methyl-propanol buffer, pH 10.2 + magnesium chloride). The resulting solution was incubated for 10 min at 37°C, and then 2.5 mL of a mixture of 250 mmol/L sodium hydroxide and 94 mmol/L sodium carbonate (color reagent) in a ratio of 1:1 was added. Thereafter, the mixture was vigorously vortexed, and the absorbance read at 590 nm using an ultraviolet–visible (UV) Spectrophotometer 7521(D) (Axiom Medical Ltd., UK); the enzyme activity was expressed as IU/L.

#### Determination of G-6-PDH activity

Glucose-6-phosphate dehydrogenase activity was determined using the manufacturer’s instructions. Buffer was added into a test tube containing 0.05 mL of nicotinamide adenine dinucleotide phosphate; 0.25 mL of plasma was then added, thoroughly mixed, and incubated for 10 min at 37°C. Then, 0.025 mL of the substrate was added and mixed. Subsequently, the absorbance was read at 340 nm for 3 min at 1 min intervals using a UV Spectrophotometer 7521, and the enzyme activity was expressed as MU/mL.

#### Determination of LDH activity

Lactate dehydrogenase activity was determined in accordance with the manufacturer’s instructions. Briefly, five volumes of 4-amino methyl propanol and lithium lactate were mixed with 1 volume of nicotinamide adenine dinucleotide as the working reagent. Approximately 1 mL of the working reagent was added to 0.02 mL of the sample and incubated at 37°C for 90 s. The absorbance was read at 340 nm for 3 min at 1 min intervals using a UV Spectrophotometer 7521, and the enzyme activity was expressed as U/L.

#### Determination of ACP activity

Total ACP activity was determined according to the manufacturer’s instructions. A mixture of 1-naphthyl phosphate and fast red TR salt was reconstituted with 10 mL of citrate buffer as the working reagent. Then, 100 mL of the working reagent was added to 200 mL of the sample, mixed thoroughly, and incubated for 5 min at 35°C. The absorbance was monitored at 405 nm for 3 min at 1 min intervals using a UV Spectrophotometer 7521; the enzyme activity was expressed as U/L.

### Inflammatory biomarkers

#### Determination of nitrite concentration

The level of nitrite was estimated as a gauge of generation of NO in accordance with the Griess method reported by Green *et al*. [41]. Briefly, Griess reagent was prepared by adding equal volumes of 0.1% N-(1-napththyl) ethylenediaminedihydrochloride and 1% sulfanilamide (dissolved in 5% phosphoric acid) [41]. Then, 50 μL of 2× distilled water-diluted supernatant was added to wells in a microtiter plate and incubated with 100 μL of Griess reagent at 25°C for 10 min away from light [41]. A standard curve of sodium nitrite (0–100 mM) was prepared for the estimation of nitrite in the sample. The absorbance was read at 540 nm using a microplate reader (LT4500, UK). The level of nitrite (in μmoles/mg protein) in the samples was estimated from the prepared standard curve.

#### Determination of IL-6 and TNF-α concentration

The levels of IL-6 and TNF-α in testicular tissue were determined by ELISA specific to the cytokines of interest, with a sensitivity minimum of 4 pg/mL, according to the manufacturer’s manual (BioLegendInc., San Diego, USA). This procedure was performed at 25°C using a microplate reader with a 450 nm filter. The concentrations of IL-6 and TNF-α in the testicular tissue were interpolated from the standard graphs of IL-6 and TNF-α and expressed as pg/mL.

### Sperm count and biological characteristics

After the removal of the epididymis, the inherent caudal fluid was obtained for analysis. Sperm motility was assessed, as reported by Khatun *et al*. [42]. Briefly, the sperm was milked on an already warmed slide, followed by the addition of approximately two drops of tepid sodium citrate (2.9%). A cover slip was added, and the mixture was assessed using a microscope (×40 objective with reduced light, Olympus CH; Olympus Japan). The viability of sperm (live/dead ratio) and the number of spermatozoa exhibiting forward unidirectional movement in a pre-determined section of the slide were observed with the aid of a light microscope with an attached camera. Spermatozoa were then stained with eosin/nigrosin dye. The sample used for epididymal sperm motility was recovered and the coverslip was removed swiftly; then two drops of the eosin/nigrosin stain were added and a smear was prepared, air-dried, and then analyzed under the microscope (×40 objective). The viable spermatozoa should not be stained (as a result of their intact cell membrane), while dead sperm cells should be stained (as a result of the compromised cell membrane). Approximately 100 sperm/slide were counted to generate the percentage of live and dead cells [42–44]. A thin smear of the sperm sample was formed on a clean slide, treated with 95% ethanol, and air-dried for sperm morphological study. Thereafter, the fixed slide was serially immersed in increasing concentrations of ethanol and stained for 6 s with Harris hematoxylin, G-6 orange stain, and EA-50 green stain. The slide was then viewed microscopically at 1000×, and 200 sperm were analyzed, with abnormalities in percentages expressed [45, 46]. The caudal epididymis was immersed in 5 mL saline solution in a graduated test tube for the sperm count, and the volume of fluid displaced was estimated as the volume of the epididymis [45]. Sperm was counted using the improved Neubauer Chamber after the caudal epididymis was homogenized in formal saline (LABART, Germany). The sperm count was conducted using a light microscope (40×) while analyzing various areas. The sperm count was given in millions per liter of suspension.

### Statistical analysis

All experiments were performed in triplicate, and GraphPad Prism version 5.0 was used to perform a one-way analysis of variance. Thereafter, Dunnett’s and Tukey’s multiple comparison post-tests were employed. At p < 0.05, the difference between means was considered significant.

## Results

### Phytochemicals in aqueous extract of BL

The phytochemicals in the aqueous extract of BL were screened and the results are presented in Table 1. This screening identified a wide range of phytochemicals, including flavonoids, phenolic compounds, alkaloids, tannin, glycosides, saponin, anthraquinones, and steroids. Of these, alkaloids and glycosides were the most abundant in the extract, whereas flavonoids, tannins, and steroids were only present in trace amounts.

**Table-1:** Phytochemicals in aqueous extract from bay leaf (*Laurus nobilis*).

Phytochemicals	Qualitative remarks
Flavonoids	+
Phenolic compounds	++
Alkaloid	+++
Tannin	+
Glycoside	+++
Saponin	++
Anthraquinones	++
Steroids	+

+++ = Heavily present, ++ = Present, + = Trace

### Effect of BL aqueous extract on growth and gonadosomatic index (GSI) of rats

The effect of BL aqueous extract on the growth and GSI of rats is shown in Table 2. There was no significant (p *>* 0.05) difference between the final and initial average weights of rats in all the experimental groups except the control, where a significant increase in the average weight of rats was observed; however, the final average weight of rats treated with AlCl_3_ did decrease, although not significantly. There was a significant (p *<* 0.05) increase in the GSI of groups treated with AlCl_3_ when compared with that of the control group. Conversely, the GSI of groups co-administered AlCl_3_ and BL extract at both concentrations (125 mg/kg BL and 250 mg/kg BL) were not significantly (p *>* 0.05) different from that of the control.

**Table-2:** Effect of BL (*Laurus nobilis*) aqueous extract on growth and GSI of rats.

Variable	Control	AlCl_3_	AlCl_3_+125mg/kg BL	AlCl_3_+250mg/kg BL
Final body weight (g)	217.3 ± 37.00^ab^	154.5 ± 22.81^ab^	135.5 ± 15.80^ab^	163.3 ± 18.28^ab^
Initial body weight (g)	156.0 ± 4.97^bc^	174.3 ± 7.14^ab^	138.8 ± 9.43^ab^	178.5 ± 6.14^ab^
GSI (%)	1.16 ± 0.19	1.70 ± 0.24^b^	1.50 ± 0.16^a^	1.27 ± 0.15^a^

Values represent mean ± standard deviation (n = 6). Values with the same superscript letter “a” along GSI row are not significantly (p > 0.05) different from control. Furthermore, values with the same superscript letters “ab” along the same column are not significantly (p > 0.05) different while the value with superscript letters “bc” is significantly (p < 0.05) different from control. GSI = Gonadosomatic index, BL = Bay leaf, AlCl_3_ = Aluminum chloride

### Effect of BL aqueous extract on testicular biomarkers

#### Alkaline phosphatase activity

**Figure-1:**
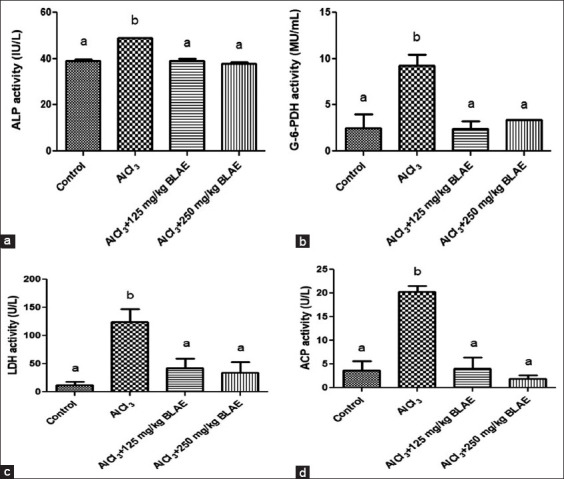
Effect of bay leaf (*Laurus nobilis*) aqueous extract on testicular function biomarkers: (a) ALP activity, (b) G-6-PDH activity, (c) LDH activity, and (d) ACP activity. ALP = Alkaline phosphatase, LDH = Lactate dehydrogenase, ACP = Acid phosphatase.

The effect of BL aqueous extract on testicular biomarkers: ALP, G-6-PDH, LDH, and ACP are presented in Figures-1a–d. There was a significant (p *<* 0.05) increase in plasma ALP activity of the group treated with AlCl_3_ relative to the control. However, there was no significant (p *>* 0.05) difference in plasma ALP activity of groups co-administered with AlCl_3_ and BL extract at low (125 mg/kg BL) or high (250 mg/kg BL) concentrations when compared with that in control (Figure-1a).

#### Glucose-6-phosphate dehydrogenase activity

Glucose-6-phosphate dehydrogenase activity followed the same trend with ALP and was significantly increased in the group treated with AlCl_3_ when compared with that in control but was significantly decreased to the level of the control by the administration of BL extract at low (125 mg/kg BL) and high (250 mg/kg BL) concentrations (Figure-1b).

#### Lactate dehydrogenase activity

The effect of BL aqueous extract on LDH activity in the plasma of rats treated with AlCl_3_ is shown in Figure-1c. There was a significant (p *<* 0.05) increase in plasma LDH activity in the group treated with AlCl_3_. However, there was no significant (p *>* 0.05) difference in plasma LDH activity of groups administered with 250 mg/kg BL extract alone or when co-administered AlCl_3_ and BL extract at low (125 mg/kg BL) or high (250 mg/kg BL) concentrations when compared with that in the control.

#### Acid phosphatase activity

Figure-1d presents the effect of BL aqueous extract on ACP activity in the plasma of rats treated with AlCl_3_. The result revealed a significant (p *<* 0.05) increase in plasma ACP activity in the group treated with AlCl_3_. However, there was no significant (p *>* 0.05) difference in plasma ACP activity of groups administered with 250 mg/kg BL extract alone or when co-administered with AlCl_3_ and BL extract at low (125 mg/kg BL) or high (250 mg/kg BL) concentrations when compared with that in control.

### Effects of BL aqueous extract on inflammatory biomarkers

#### Nitrite concentration

**Figure-2:**
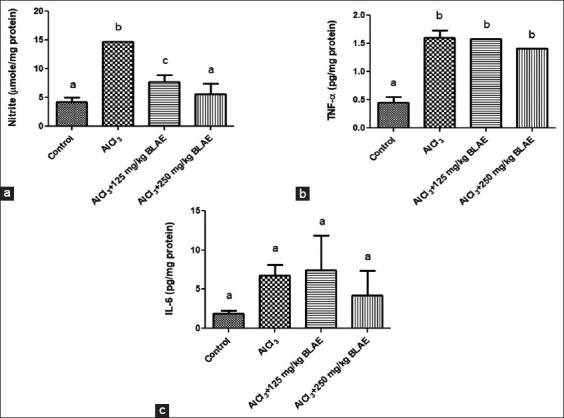
Effects of bay leaf (*Laurus nobilis*) aqueous extract on inflammatory biomarkers: (a) Nitrite, (b) TNF-α, and (c) IL-6. TNF-α = Tumor necrosis factor-alpha, IL-6 = Interleukin-6.

The effect of BL aqueous extract on nitrite levels in rats treated with AlCl_3_ is shown in Figure-2a. There was a significant (p *<* 0.05) increase in testicular nitrite concentration of the AlCl_3_ group when compared with that in the control. However, there was no significant (p *>* 0.05) difference in testicular nitrite concentration of other treatment groups (250 mg/kg BL extract, AlCl_3_ + 125 mg/kg BL extract, or AlCl_3_ + 250 mg/kg BL extract) when compared with that in control. This indicates that BL extract at low and high concentrations ameliorated the toxic effect of AlCl_3_ on the testes of rats.

#### Interleukin-6 and TNF-α concentration

Although there was a significant (p *<* 0.05) increase in testicular TNF-α concentration in all the treatment groups when compared with that in the control, (Figure-2b), there was no significant (p *>* 0.05) difference in the testicular TNF-α concentration of groups cotreated with AlCl_3_ and BL extract at either low (125 mg/kg) or high (250 mg/kg) concentrations when compared with that in the toxicant (AlCl_3_) group. Notably, the concentration of TNF-α was lowest in the AlCl_3_ + 250 mg/kg BL group when compared with that in the other treatment groups. The effect of BL aqueous extract on IL-6 concentration in rats treated with AlCl_3_ is shown in Figure-2c. There was no significant (p *>* 0.05) difference in testicular IL-6 concentration in any of the treatment groups when compared with that in control. Although the concentration of testicular IL-6 in the toxicant (AlCl_3_) group was higher than that in control, cotreatment with low (125 mg/kg) or high concentration (250 mg/kg) of BL extract did not produce significant effects.

### Effects of BL aqueous extract on sperm count and biological characteristics

**Figure-3:**
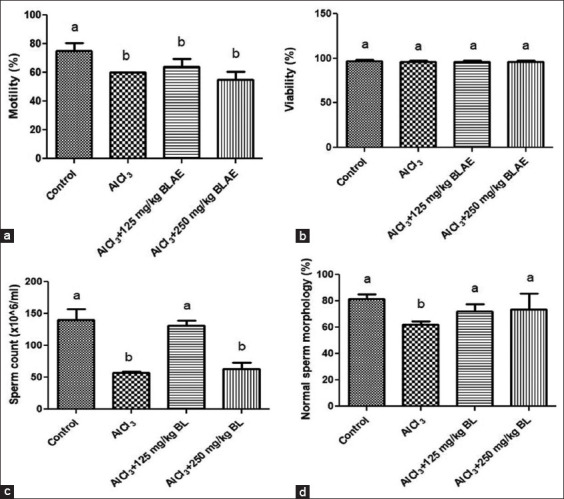
Effects of bay leaf (*Laurus nobilis*) aqueous extract on sperm biochemical characteristics: (a) Motility, (b) viability, (c) count, and (d) morphology.

The effect of BL aqueous extract on sperm motility in rats treated with AlCl_3_ is detailed in Figure-3a, which shows that there was a significant (p < 0.05) difference across the groups. Post-test analysis revealed a significant (p < 0.05) decrease in sperm motility in groups treated with AlCl_3_, AlCl_3_ + 125 mg/kg BL, and AlCl_3_ + 250 mg/kg BL extracts when compared with that in the control group. However, there was no significant (p > 0.05) difference in the sperm motility of test groups when compared with that in the AlCl_3_ group. The effect of BL aqueous extract on sperm viability in rats treated with AlCl_3_ is presented in Figure-3 band shows that there was no significant (p > 0.05) difference in sperm viability of groups treated with AlCl_3_, AlCl_3_ + 125 mg/kg BL, or AlCl_3_+250 mg/kg BL extract when compared with that in the control group. This indicated that administration of AlCl_3_ and BL extract at low and high concentrations did not affect sperm viability. The effect of the BL extract on sperm counts in rats treated with AlCl_3_ is presented in Figure3c and shows a significant (p *<* 0.05) decrease in sperm count of groups treated with AlCl_3_ and AlCl_3_ + 250 mg/kg BL extract. However, there was no significant (p *>* 0.05) difference in the sperm count of groups treated with AlCl_3_ + 125 mg/kg BL extract when compared with that in the control group. Importantly, co-administration of BL extract at a higher dose (250 mg/kg BW) with AlCl_3_ may potentiate the effect of AlCl_3_ on sperm count. Furthermore, Figure-3d reveals a significant (p *<* 0.05) decrease in normal sperm morphology in the group administered with AlCl_3_ when compared with that in control, whereas co-treatment with BL extract at both doses (125 and 250 mg/kg BW) increased the percentage of sperm with normal morphology compared with that in the control group.

## Discussion

*Laurus nobilis*-bay leaf possesses several medicinal effects such as wound healing, antioxidant, antibacterial, antiviral, immunostimulant, anticholinergic, antifungal, insect repellant, anticonvulsant, antimutagenic, analgesic, and anti-inflammatory activities [47]. This study investigated the effects of aqueous extract of *L. nobilis* on testicular function biomarkers (ALP, G-6-PDH, LDH, and ACP), several inflammatory biomarkers (nitrite, TNF-α, and IL-6), and sperm biochemical characteristics (sperm motility, sperm viability, sperm count, and sperm morphology) in male rats exposed to AlCl_3_.

The biochemical parameters examined in this study are relevant testicular function markers [48]. The toxic effect of a compound/plant extract on tissues may be determined by measuring the activity of testicular function enzymes in tissues and plasma/serum [49] and can be used as an indicator of tissue cellular damage caused by a chemical compound before histological changes are detected [48]. The increase in plasma activity of testicular enzymes (ALP, G-6-PDH, LDH, and ACP) in animals treated with AlCl_3_ (30 mg/kg BW) indicated testicular damage induction in this group, which probably led to the leakage of the enzymes into the blood. However, co-administration of BL aqueous extract with AlCl_3_ at the two doses used (125 and 250 mg/kg BW) restored the plasma activity of the testicular enzymes (ALP, G-6-PDH, LDH, and ACP) to the level of the control group, suggesting the ameliorative effect of the extract against AlCl_3_-induced testicular damage. This suggests that the extract may not be toxic to the testes of the rats at these doses. These findings agree with those of Akunna *et al*. [50], who reported that *L. nobilis* extract preserved testicular functions in cryptorchid rats. Similarly, Marza *et al*. [51] reported the protective effect of *L. nobilis* aqueous extract against AlCl_3_-induced liver toxicity in rats.

Furthermore, the BL aqueous extract did not have any significant effect on TNF-α and IL-6 levels but did inhibit nitrite production, thereby suggesting an anti-inflammatory activity *in vivo*. Increased development of a highly toxic anion of peroxidation (peroxynitrite ONOO) induces testis degeneration under specific pathological states, such as increased NO production [52]. Nitrite is often employed as a surrogate biomarker of NO production, which is important in the etiology of inflammation, and therefore, the elevated testicular nitrite level observed in rats administered with AlCl_3_ in this study may suggest testicular inflammation. Nitric oxide is a pro-inflammatory mediator that causes inflammation when produced in excess under abnormal conditions. Several studies reported that the nitrate-nitrite-NO cycle can act as a circulating endocrine reservoir of NO [53–55]. The reduced level of nitrite in the groups cotreated with BL extract and AlCl_3_ suggests an anti-inflammatory property through probable inhibition of NO production. This finding agrees with previous related studies that reported the anti-inflammatory property of BL *in vivo* [56, 57].

Spermatozoa are motile cells that serve as sensitive endpoints for assessing the effects of plant extracts on cellular activity [58]. Findings from this study showed that AlCl_3_ had adverse effects on most of the biological characteristics of the sperm investigated (reduced sperm motility, reduced sperm count, and decreased percentage of sperm with normal morphology), thereby confirming their protoxic effect in male rats. Nevertheless, co-treatment of rats with AlCl_3_ and BL aqueous extracts at both doses (125 mg/kg and 250 mg/kg BW) did not have a significant effect on sperm motility. Similarly, the co-treatment with AlCl_3_ and BL extracts did not affect sperm viability in this study. The effect of BL aqueous extract was most pronounced on sperm count when co-administration with AlCl_3_ at a low dose (125 mg/kg BW) and improved sperm count in treated rats, whereas co-administration of BL aqueous extract with AlCl_3_ at a high dose (250 mg/kg BW) may potentiate the toxic effect of AlCl_3_ on sperm count, indicating that BL aqueous extract ameliorated the toxic effect of a low dose of AlCl_3_ on sperm count. Interestingly, combining AlCl_3_ with BL aqueous extract enhanced the percentage of sperm with normal morphology. This study shows that orally administering an aqueous extract of BL enhanced sperm quality in rats treated with AlCl_3_, which is consistent with the findings of Akunna *et al*. [59], who found a significant improvement in all sperm parameters in varicocele rats treated with BL. Considering the significance of sperm count and sperm morphology in male fertility in this study, it is possible that BL aqueous extract may help to improve sperm quality, thereby enhancing male fertility.

The bioactivity of BL aqueous extract in this study may be linked to the presence of different classes of phytochemicals, including flavonoids, phenolic compounds, alkaloids, tannin, glycosides, saponin, anthraquinones, and steroids as highlighted in our previous study [34]. The identified groups of compounds in the extract are characterized by significant pharmacological properties, including antioxidant and anti-inflammatory effects [60, 61]. Alkaloids and glycosides have been reported to exhibit anti-inflammatory activity by inhibiting pro-inflammatory cytokines [61]. Similarly, flavonoids and other phenolics have displayed significant antioxidant and anti-inflammatory properties [62]. Alkaloids, which were the most abundant phytochemicals in the BL extract, are notable antioxidants and can inhibit iron-induced lipid peroxidation stimulated by aluminum [63, 64]. This is corroborated by Khodja *et al*. [65], who reported the antioxidant properties of alkaloid extract from BL. Moreover, one study has reported that the identified constituents of the extract are essential for sperm quality [46].

## Conclusion

The study revealed that BL aqueous extract at low and high doses ameliorated the damaging effect of AlCl_3_ on the testes of rats. The BL extract exhibited an anti-inflammatory property in AlCl_3_- treated male rats through a nitrite inhibition mechanism, and a low concentration of the BL extract improved sperm counts in rats. However, a high concentration of the extract could potentiate the toxic effect of AlCl_3_ on sperm count. The anti-inflammatory property and ameliorative effect of BL aqueous extract against AlCl_3_-induced reproductive toxicity in male rats could be due to the presence of a wide range of phytochemicals, particularly alkaloids and glycosides. Therefore, BL aqueous extract may be a promising source of novel compounds with male fertility-promoting and anti-inflammatory properties that could be exploited for further study.

## Authors’ Contributions

AOF and KEA: Conceptualized the study. AOA and HAI: Conducted the experiment. AOF, KEA, AOA, and HAI: Drafted the manuscript. AOF, KEA, AOA, HAI, KO, and OOO: Proofread and edited the manuscript. All authors have read and approved the final manuscript.
